# Anxiety and error monitoring: the importance of motivation and emotion

**DOI:** 10.3389/fnhum.2013.00636

**Published:** 2013-10-08

**Authors:** Greg H. Proudfit, Michael Inzlicht, Douglas S. Mennin

**Affiliations:** ^1^Department of Psychology, Stony Brook University, Stony Brook, NY, USA; ^2^Department of Psychology, University of Toronto, Toronto, ON, Canada; ^3^Department of Psychology, Hunter College, City University of New York, New York, NY, USA

**Keywords:** ERN, anxiety, endophenotype, error-related negativity, threat

## Introduction

Moser et al. (2013) report a novel meta-analysis across 37 studies demonstrating a small-to-medium association between the error-related negativity (ERN) and self-report measures of anxiety (*r* = −0.25); the meta-analysis further indicates a stronger relationship between the ERN and anxious apprehension (*r* = −0.35) than anxious arousal (*r* = −0.09). Based on these results, Moser et al. articulate their *compensatory error monitoring hypothesis (CEMH)*. In brief, the CEMH proposes that the relationship between anxious apprehension and an increased ERN is due to the distracting effects of worry: worrisome thoughts make it more difficult for anxious individuals to maintain task-related goals; as a result, increased effort must be employed. The CEMH suggests that an increased ERN reflects the transient increase in effort to compensate for the distracting effects of worry. Though we agree with many aspects of the CEMH (e.g., the importance of apprehensive anxiety; the potential impact of worry on the ERN), we believe that motivation and emotion are central constructs to understanding both within- and between-subjects variation in the ERN.

## Errors are aversive (especially for anxious people)

Threat has traditionally been conceptualized in terms of external stimuli—things with the capacity or intention to harm an individual. We hypothesized that the commission of errors might similarly be threatening (Hajcak and Foti, 2008; Hajcak, 2012): making mistakes place an individual in unknown danger. In support of this view, errors are experienced as distressing (Spunt et al., 2012) and are associated with a host of physiological changes consistent with defensive mobilization: following errors, the startle reflex is increased (Hajcak and Foti, 2008; Riesel et al., 2013), heart rate decelerates (Hajcak et al., 2003, 2004), the pupil dilates (Critchley et al., 2005), the corrugator (i.e., frowning) muscle contracts (Lindstrom et al., 2013), and a sympathetic nervous system response is evident in skin conductance changes (Hajcak et al., 2003, 2004). Moreover, there is increasing behavioral evidence that errors and other variants of response conflict are aversive (Botvinick, 2007; Dreisbach and Fischer, 2012; Schouppe et al., 2012). Indeed, errors activate many of the same neural circuits associated with the experience of negative affect (Shackman et al., 2011).

Previously we used the term *defensive motivation* in discussing both state and trait effects (Hajcak, 2012; Weinberg et al., 2012b); to avoid potential confusion here, we use the term *threat sensitivity* to refer to trait-like individual differences which we contrast with *defensive motivation*, which reflects a transient response to threat. Thus, we view errors as unpredictable threats that prompt an immediate defensive motivational response. Further, we believe that variation in the ERN reflects a *trait* difference in early *threat sensitivity* that drives vigilance and increased defensive motivational responses. This view is consonant with theories of early-emerging and stable individual differences in temperamental styles such as behavioral inhibition (Fox et al., 2005) and related forms of dispositional anxiety (Fox et al., 2008; Shankman et al., 2013). High behavioral inhibition describes increased sensitivity to environmental cues of punishment, novelty, and threat (Gray and McNaughton, 2000); dispositional anxiety refers to a tendency to respond excessively in the face of potential or *uncertain* threats (Barlow, 2002; Grupe and Nitschke, 2013; see also Hirsh and Inzlicht, 2008).

In this context, we argue that the increased ERN characteristic of anxious individuals reflects the disposition to respond more strongly to uncertain threat (Hajcak, 2012; Weinberg et al., 2012b). Moser et al. suggest that there is no evidence that anxious individuals are characterized by a greater defensive response to errors. However, in one study, participants scoring high in trait negative emotionality demonstrated larger increases in skin conductance after making errors (Hajcak et al., 2004). Moreover, anxious people report excessive concern about their mistakes. We would similarly predict larger startle responses after errors among more anxious individuals, and would encourage additional studies in which variability in the ERN is examined in relation to other indices of threat sensitivity and defensive motivation.

## The ERN as endophenotype

We (Olvet and Hajcak, 2008) and others more recently (Manoach and Agam, 2013) have argued that there is considerable evidence that the ERN is a candidate psychiatric endophenotype. An endophenotype must be associated with an illness, heritable, evident in unaffected first-degree family members, and independent of current disease state (Gottesman and Gould, 2003; Miller and Rockstroh, 2013). Moser et al. dismiss this possibility, citing only one study in which treatment-related reductions in OCD symptoms did not reduce the ERN in a pediatric Obsessive-Compulsive Disorder (OCD) sample (Hajcak et al., 2008). However, there is considerable evidence that the ERN is stable over time and behaves like an endophenotype. For instance, the ERN demonstrates sufficient test-retest reliability over two weeks to more than two years (Olvet and Hajcak, 2009; Weinberg and Hajcak, 2011). Moreover, about 50% of the variation in ERN amplitude appears to be heritable (Anokhin et al., 2008), and variation in the ERN has been linked to a variety of genes (Manoach and Agam, 2013). Two recent studies found an increased ERN in unaffected first-degree relatives of OCD patients (Riesel et al., 2011; Carrasco et al., 2013). These data point toward the ERN as a neural endophenotype.

## Distinguishing reactivity to threat from subsequent compensatory processes

A fundamental distinction between our view and the CEMH is that we do not view the relationship between ERN and anxiety as compensatory. We make a strong distinction between temporally earlier defensive motivational responses that vary with threat sensitivity and later compensatory responses that include cognitive processes such as worry (Borkovec et al., 2004; Newman and Llera, 2011; Mennin and Fresco, 2013). We believe that increased threat sensitivity (i.e., behavioral inhibition) precedes the development of compensatory processes such as worry—both phylogenetically and ontogenetically. That is, heightened trait differences in threat sensitivity can lead to various forms of cognitive compensation—including worry. As such, we would argue that worriers actually have two problems: they are more sensitive to uncertain threat, and they have developed maladaptive cognitive coping strategies to deal with their increased threat sensitivity (e.g., worry).

Within this framework, we believe that the ERN relates to trait-like vulnerabilities in threat sensitivity rather than compensatory efforts to modulate increased threat sensitivity such as worry. This distinction is especially relevant in terms of prospective and developmental predictions. For instance, a formal worry process may not be clearly evident in young children (Vasey et al., 1994). However, we found increased ERNs in clinically anxious 6 year-olds, who were mainly phobic (Meyer et al., 2013). This would suggest that increased ERN, reflecting heightened threat sensitivity, develops *before* processes like worry. Our model presumes that an increased ERN would prospectively predict increases in anxiety and worry—and that an increased ERN would be a risk marker for the development and onset of anxiety disorders. Our model would also predict an increased ERN among more anxious non-human animals that are presumably less prone to verbally-mediated compensatory processes such as worry; for instance, the ERN can be measured in non-human primates (Godlove et al., 2011) who show marked differences in behavioral inhibition and anxiety (Fox et al., 2008).

## The ERN is sensitive to state affect

Many trait-like measures and phenotypes (e.g., anhedonia) can be altered and manipulated in the short-term (e.g., via stressors and mood inductions; also see Coan et al., 2006). Moser et al. argue that changes in state affect do not consistently modulate the ERN. However, it might be important to distinguish between affect that is integrally related to errors and affect that is incidental (see Schmeichel and Inzlicht, 2013). When spider phobics make errors on a flanker task in the presence of a spider, their fear is incidental to error processing (Moser et al., 2005). However, if their fear was related to making an error (e.g., if spider phobics had to view pictures of spiders after making mistakes), then their fear would be integrally related to errors. Emerging data suggests that variation in motivation to make errors does impact the ERN. When integral negative affect is added, such as when errors are punished (Riesel et al., 2012), when performance is evaluated (Hajcak et al., 2005), when errors are more valuable (Hajcak et al., 2005) or personally meaningful (Amodio et al., 2008; Legault and Inzlicht, 2013), the ERN tends to increase; when integral negative affect is subtracted, such as when people are led to misattribute their affect to an external and benign source (Inzlicht and Al-Khindi, 2012) or when they ingest an anxiolytic agent that leads them to care less about their errors (Bartholow et al., 2012), the ERN decreases. In our model, worrying might potentiate the ERN if it were to increase the threat value of errors. It will be important for future studies to determine the extent to which state variability in worry accounts for the relationship between trait anxiety and the ERN.

## Future challenges

Moser et al.'s paper encourage greater phenotypic specificity for understanding the increased ERN in relation to anxiety—and this is a significant contribution (see also Vaidyanathan et al., 2012). Worry is one phenotype that *may* account for the increased ERN in anxiety disorders; however, we would also encourage continued efforts to evaluate the ERN in relation to additional, empirically-derived phenotypes (Watson et al., 2007). Indeed, some extant clinical data already suggests that the relationship between anxiety and the ERN may require examining the *interaction* between key phenotypes. For instance, comorbid major depressive disorder (MDD)—which is also characterized by increased worry—appears to mask the relationship between GAD and an increased ERN; history of MDD, however, does not seem to impact the increased ERN in GAD (Weinberg et al., 2012a). We have suggested that *state*-related characteristics of depression (i.e., anhedonia) may alter the relationship between ERN and *trait* anxiety.

Moser et al. also sound a call for more specific predictions and assertions regarding the relationship between ERN and anxiety. We agree, and our view focuses on possible causes and subsequent development of anxiety disorders (i.e., models of etiopathogenesis). One possibility from the endophenotype perspective is that the same genes that confer risk for the development of anxiety disorders determine variability in the ERN. Another possibility is that environmental (i.e., non-genetic) factors that impact error salience modulate the ERN. In an approach rooted in models of fear conditioning and extinction-based learning, we inflated the threat value of errors by punishing certain mistakes; even after mistakes were no longer punished, the ERN was potentiated on trials that had formerly been punished (Riesel et al., 2012). Based on these data, we suggested that early learning experiences (e.g., critical parenting) may lead to a larger ERN. Our view is consonant with the possibility that ERN neurodevelopment is impacted by both genetic and environmental factors that shape characteristic defensive motivational responses to errors. There are multiple pathways to increased threat sensitivity.

Our conceptualization gets at a fundamental issue: why are some people more worried to begin with? Our view is that an elevated ERN reflects a broad disposition toward increased sensitivity to uncertain threat, and that some individuals attempt to compensate for this via worry (Mennin and Fresco, 2013). The most significant advantages of the endophenotype approach are the potential for identifying genetic contributions to disorders (e.g., the genetics of the ERN are simpler than the genetics of complex disorder-based phenotypes), for identifying those at risk for disorders, and for bridging human and animal models. Future steps would then include more mechanistic studies to clarify causation and identify novel interventions. Accordingly, we suggest further research to understand the conditions under which variability in the ERN *leads* to pathological outcomes. Could manipulating the ERN causally alter risk for anxiety and compensatory efforts like worry? As a proposed metaphor, we consider the relationship between cholesterol and coronary heart disease (CHD): high levels of low density lipoproteins (LDL) is a partially inherited risk factor for CHD; risk for CHD is lowered by directly manipulating LDL through medication and lifestyle change. LDL levels are trait-like, genetically determined, and yet, are sensitive to state-related (i.e., diet) manipulations; lowering LDL alters subsequent risk for disease. In this way, the ERN itself might be a unique target for intervention and prevention efforts. Our view focuses on leveraging variability in the ERN to understand the development of, and risk for, psychological disorders. This approach requires large and longitudinal studies to delineate trajectories of risk, and to parse the prospective relationship between ERN and increases in anxiety.

## Conclusion

Although there is much to like about the CEMH, we believe that it does not fully address the critical contribution of emotion to the ERN. Importantly, when examining the influence of anxiety on the ERN, it is vital to account for trait-level differences in emotionality; to distinguish between threat sensitivity and compensatory efforts to deal with threat such as worry, and to differentiate between integral and incidental affect. Emotion is both a core aspect of anxiety and why errors powerfully shape behavior. Emotion is at the heart of the anxiety-ERN relationship.
